# Transformer-Based Multi-Modal Data Fusion Method for COPD Classification and Physiological and Biochemical Indicators Identification

**DOI:** 10.3390/biom13091391

**Published:** 2023-09-15

**Authors:** Weidong Xie, Yushan Fang, Guicheng Yang, Kun Yu, Wei Li

**Affiliations:** 1 School of Computer Science and Engineering, Northeastern University, Hunnan District, Shenyang 110169, China; 1910638@stu.neu.edu.cn (W.X.); fyswork@163.com (Y.F.); 2272111@stu.neu.edu.cn (G.Y.); 2 College of Medicine and Bioinformation Engineering, Northeastern University, Hunnan District, Shenyang 110169, China; yukun@bmie.neu.edu.cn; 3 Key Laboratory of Intelligent Computing in Medical Image (MIIC), Hunnan District, Shenyang 110169, China

**Keywords:** multi-modal fusion, cross-modal transformer, low-rank multi-modal fusion, COPD

## Abstract

As the number of modalities in biomedical data continues to increase, the significance of multi-modal data becomes evident in capturing complex relationships between biological processes, thereby complementing disease classification. However, the current multi-modal fusion methods for biomedical data require more effective exploitation of intra- and inter-modal interactions, and the application of powerful fusion methods to biomedical data is relatively rare. In this paper, we propose a novel multi-modal data fusion method that addresses these limitations. Our proposed method utilizes a graph neural network and a 3D convolutional network to identify intra-modal relationships. By doing so, we can extract meaningful features from each modality, preserving crucial information. To fuse information from different modalities, we employ the Low-rank Multi-modal Fusion method, which effectively integrates multiple modalities while reducing noise and redundancy. Additionally, our method incorporates the Cross-modal Transformer to automatically learn relationships between different modalities, facilitating enhanced information exchange and representation. We validate the effectiveness of our proposed method using lung CT imaging data and physiological and biochemical data obtained from patients diagnosed with Chronic Obstructive Pulmonary Disease (COPD). Our method demonstrates superior performance compared to various fusion methods and their variants in terms of disease classification accuracy.

## 1. Introduction

Advances in biomedical technology allow researchers to access rich data on different modalities of the same disease, such as mRNA expression data, DNA methylation data, microRNA (miRNA) expression data, physiological and biochemical data, Computed Tomography (CT) image data, Whole Slide Image (WSI) data. Single-modality data can provide a partial picture of biological complexity, while integrating multi-modal data can provide a more comprehensive view of underlying biological processes [1]. Understanding the relationship between data using different modalities is essential for us to analyze biological processes for further classification of diseases [2]. In addition, multi-modal fusion can effectively utilize the information overlooked by a single modality [3].

In biomedical data analytics, advancements in data fusion techniques are heralding transformative shifts. These techniques are primarily tailored to consolidate multifaceted data sources, revealing implicit biological knowledge and patterns. The integration of multi-omics seeks to encompass information spanning various molecular strata, inclusive of gene expression, proteomics, and metabolomics, thereby bestowing a comprehensive perspective on the mechanisms underlying disease onset [4]. Concurrently, the multi-modal fusion methodologies endeavor to unify data stemming from divergent technological platforms, such as gene expression datasets fushion with medical imaging data to derive enhanced accuracy in characterizing disease features and in the formulation of predictive models [5].

Several remarkable multi-modal data fusion methods have been reported in the context of biomedical data. For instance, Günther et al. proposed a computational multi-modal fusion approach that effectively combined biomarkers from genomics and proteomics data, as demonstrated in their study [6]. In another study, Sun et al. enhanced breast cancer prognosis by integrating clinical and genetic markers, resulting in improved predictive accuracy [7]. Wang et al. developed a similarity network fusion method that successfully fused mRNA expression, DNA methylation, and miRNA expression data, demonstrating its effectiveness across five different diseases [8]. Additionally, Wiel et al. introduced an adaptive group regularization approach to fuse multi-modal data, yielding improved prediction accuracy [9]. In the domain of prognostic analysis for clear cell renal cell carcinoma (ccRCC), Ning et al. designed a cross-modal feature fusion approach that effectively integrated expression data, CT imaging data, and WSI imaging data, enabling accurate prognostic analysis [10].

However, it is worth noting that these existing approaches do not fully exploit the complementary nature of multi-modal data information. While feature-level fusion can enhance model accuracy, there exist dependencies and complementary relationships between attributes from different modalities or even within the same modality. Exploiting these relationships can improve learning ability and flexibility. For instance, Jl et al. employed a probabilistic graphical model-based approach, leveraging information entropy and conditional probability, to infer interactions between features for biomarker selection and disease prediction using Markov decision processes [11]. Saranya et al. successfully utilized random forest feature sensitivity and feature correlation for heart disease prediction [12]. On multi-modal data, Huang et al. applied multi-omics neural networks for survival analysis in breast cancer [13]. Wang et al. proposed Multi-Omics Graph cOnvolutional NETworks (MOGONET), which employed graph neural networks to analyze the relationships among three expression data types and implemented feature fusion using the View Correlation Discovery Network (VCDN) [14].

While these methods capture interactions and complementary information within or between modalities, they still need to address the challenge of capturing long-term and remote dependencies across modal elements and improving the adaptation between multi-modal fusion and unimodality [15]. In recent years, multi-modal fusion methods such as Low Rank Matrix Factorization (LMF) [16] and Multi-modal Transformers (MuIT) [17] have emerged as effective approaches for tensor fusion, enabling the capture of interactions between multiple modalities and potential adaptation relationships. However, to the best of our knowledge, these methods have yet to be effectively applied to biomedical data.

In response to these challenges, we have developed a novel multi-modal fusion model that effectively captures intra- and inter-modal interactions and adaptive relationships within multi-modal biomedical data. Our model aims to address disease classification and feature selection tasks. To process the expression data, we employ a graph neural network, building upon our previous research [18,19]. This network is capable of capturing feature dependencies and performing information representation. Simultaneously, we utilize a 3D convolutional network to process the image data, capturing relationships between different layers of the data.

The multi-modal vectors obtained from the expression and image data are fused using Low Rank Matrix Factorization. Subsequently, Cross-modal Transformers are employed to further enhance the complementarity between modalities and facilitate adaptation between them. Notably, our graph neural network-based approach for processing expression data goes beyond feature extraction, allowing for feature selection through pooling techniques. To the best of our knowledge, this is the first attempt in the field of biomedical data to leverage graph neural networks and 3D convolutional networks for multi-modal data processing, as well as the first application of LMF and Cross-modal Transformers for biomedical data fusion.

To evaluate the effectiveness of our proposed method, we conducted comprehensive experiments on lung CT imaging data and Physiological and Biochemical (PB) data obtained from patients with Chronic Obstructive Pulmonary Disease. The results highlight the superiority of multi-modal data integration over a single modality approach. Moreover, we performed extensive ablation experiments, demonstrating the necessity of employing graph neural networks, 3D convolutional networks, LMF, and Cross-modal Transformers. Additionally, we conducted an analysis of the physiological and biochemical indicators obtained using our proposed method, showcasing its potential for important indicators identification. The main contributions of this paper are summarized as follows.

- The first application of graph neural networks (to process expression data) and 3D convolutional networks (to process image data) in the biomedical field, effectively capturing feature dependencies and inter-layer relationships.
- Advanced multi-modal data fusion through low-rank matrix factorisation (LMF) with cross-modal transformers to enhance inter-modal complementarity.
- This study is the first attempt to use graph neural networks and 3D convolutional networks for biomedical multi-modal data processing, as well as the first application of LMF and cross-modal transformers in biomedical data fusion.
- In addition to demonstrating their superiority in multi-modal data integration, ablation experiments validate the necessity of each component of the model and highlight their potential for key metric identification.

## 2. Dataset and Experimental Setup

### 2.1. Dataset

The dataset used in this study consists of CT images and PB data, specifically focusing on lung CT images of COPD patients. The CT images were collected from Shengjing Hospital of China Medical University, including a total of 470 lung CT images. Among the patients, there were 279 males and 191 females, with an average age of 71 years. The CT scans were conducted using specific parameters, such as a kVp of 120 KV, an X-ray tube current of 202.8 ± 77.9 (mA), and a pixel size of 0.722 ± 0.057 (mm). The slice thickness varied between 1, 3, and 5 mm, corresponding to a total of 71, 174, and 225 images, respectively. For this study, the CT images were reconstructed using smooth reconstruction kernels, such as Siemens B31f or Philips B.

Additionally, the dataset included physiological and biochemical data from 295 patients. This data consisted of seven physiological indicators, namely age, sex, height, weight, smoking status, positive diastolic test results, and BMI. Furthermore, there were 17 biochemical indicators, which are listed in Table 1. All 295 cases with corresponding physiological and biochemical data were used for the experiments conducted in this paper. The diagnosis of patients was based on pulmonary function test reports and clinical diagnoses made by experienced physicians. Based on the pulmonary function indicators, patients were classified into two stages: early stage (GOLD 1–2) and advanced stage (GOLD 3–4), representing different severity levels of COPD.

### 2.2. Data Pre-Processing Methods

During the preprocessing phase, several steps were undertaken for handling abnormal values and preparing the data for analysis.

Physiological and Biochemical Data: Abnormal values in the physiological and biochemical data were addressed using the 3σ principle, which involves identifying values that deviate by more than three standard deviations from the mean, In our study, approximately 1.67% of the input data was identified as outliers. These abnormal values were then treated and filled using the K-nearest neighbors method. We utilized the KNNImputer implementation from the Scikit-learn, setting K to 10 and constraining the search exclusively to samples of the same category. Given that the COPD dataset employed in this study is characterized by a limited number of samples and features, direct exclusion of these data points might lead to the potential loss of valuable information. Thus, in scenarios where the proportion of outliers is minimal, we opted for imputation techniques to preserve the overarching structure and patterns inherent in the data more effectively. Additionally, for multi-valued data where patients underwent the same laboratory test multiple times during a visit, the least squares method was employed to address the issue of multiple values. The physiological and biochemical indicators for the day the lung function test was performed were calculated using the correlations between these multiple values.

Image Data: Preprocessing steps for the image data primarily included lung parenchyma segmentation, Housesfield Unit (HU) value normalization, axial rotation, cropping, and size normalization. The U-Net pre-training model, specifically the Unet_R231 network [20], was utilized for lung parenchyma segmentation of COPD patients’ lung CT images. To enhance the model’s perception of lung texture, HU value normalization was performed using the equation shown in Equation (1).

(1) $$I\left(x,y\right)=\frac{H\left(x,y\right)-HU_{min}}{HU_{max}-HU_{min}}$$

where H(x,y) represents the original HU value, I(x,y) represents the normalized value after CT normalization, and $HU_{min}$ and $HU_{max}$ are the minimum and maximum HU values in the dataset, respectively.

**Figure 1:**
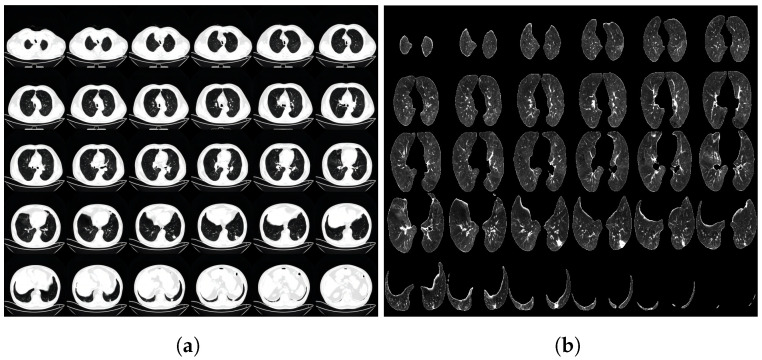
The CT image data before and after preprocessing, (**a**) is before preprocessing and (**b**) is after preprocessing.

**Figure 2:**
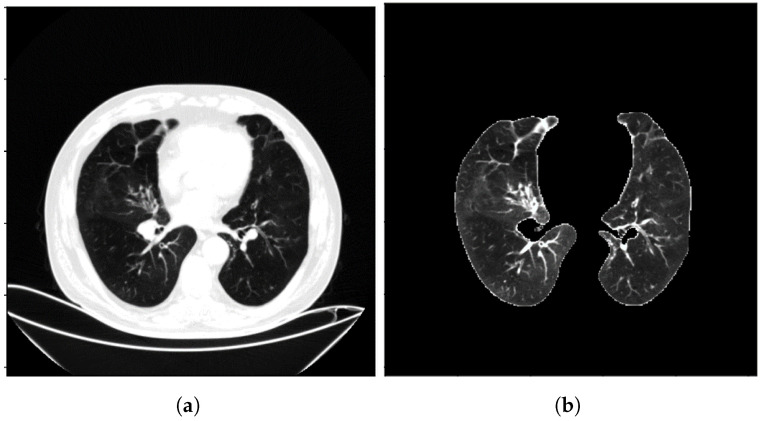
The CT image data before and after preprocessing (larger scale), (**a**) is before preprocessing and (**b**) is after preprocessing.

To increase the diversity of the samples, the 3D CT images underwent random rotations with angles within ±15${}^{\circ }$ around the *Z*-axis of the CT images. Afterward, disease-related regions that might not be easily noticed by the network were cropped, and the cropped images were resized to 224×224 pixels. For the experimental setup, the dataset was divided into a training set, validation set, and test set in a ratio of 3:1:1. This division ensured that the data were adequately represented for training, model validation, and final evaluation. To provide visual examples, Figure 1 illustrates the CT image data before and after the preprocessing steps, showcasing the impact of the normalization and cropping procedures. Figure 2 illustrates the CT image data before and after the preprocessing steps at a much larger scale.

### 2.3. Experimental Environment

The experiments were performed on a system running Ubuntu 18.04, equipped with an Intel(R) Xeon(R) Silver 4110 CPU, 128.0GB RAM, and 8 NVIDIA GTX 1080 GPUs. The implementation was carried out using Python 3.9, with the primary methods being implemented using scikit-learn 1.2.0. The PyTorch framework version 1.13.1 was utilized for implementing the main components of the proposed method, and the graph neural network was implemented using the PyTorch Geometric framework. These software tools and frameworks provided the necessary functionalities and libraries required for data processing, model training, and evaluation in the conducted experiments. In our experiments, a five-fold cross-validation was consistently employed, with the average of different evaluation metrics being considered as the final results.

### 2.4. Evaluation Metrics

The evaluation metrics employed in our experiments encompass Accuracy, Precision, Sensitivity, Specificity, F1-Score, and AUC. These metrics were chosen to quantitatively analyze the experimental results and evaluate the performance of the model. Their calculations are illustrated from Equation (2).

(2) $$\begin{array}{c} \;\mathrm{Accuracy}\;=\frac{TP+TN}{TP+TN+FP+FN}\\ \;\mathrm{Precision}\;=\frac{TP}{TP+FP}\\ \;\mathrm{Sensitivity}\;=\frac{TP}{TP+FN}\\ \mathrm{Specificity}=\frac{TN}{TN+FP}\\ F1-\mathrm{Score}=\frac{2\times \;\mathrm{Precision}\;\times \;\mathrm{Recall}\;}{\;\mathrm{Precision}\;+\;\mathrm{Recall}\;} \end{array}$$

where TP represents the number of samples that are correctly predicted as late-stage COPD. FP represents the number of samples that are incorrectly predicted as late-stage COPD but are actually early-stage COPD. FN denotes the number of samples that are incorrectly predicted as early-stage COPD but are actually late-stage COPD. TN denotes the number of samples that are correctly predicted as early-stage COPD.

## 3. The Proposed Method

### 3.1. Overall Framework of the Proposed Method

**Figure 3:**
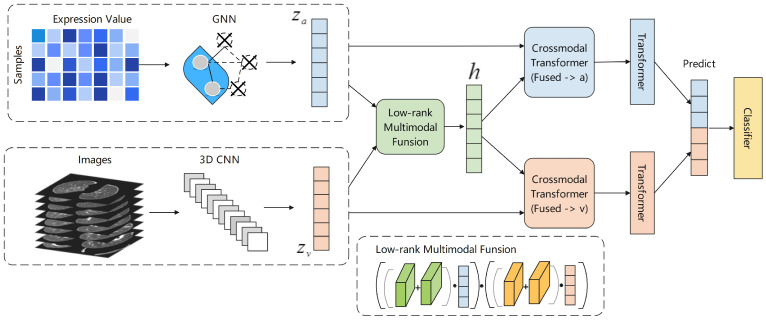
Overall process of proposed method. The GNN and 3D CNN networks are used to process the PB and image CT, respectively, to obtain the corresponding feature vectors $z_{a}$ and $z_{v}$. Subsequently, the LMF method is used to fuse the feature vectors *h*. The $z_{a}$, *h*, and $z_{v}$, *h* are input to the Cross-modal Transformer module, respectively, to obtain the corresponding fusion vectors, and after a layer of the Self-attention Transformer module, finally, the final vectors of the two modalities are stitched together.

In this subsection, we present the overall framework of our proposed method, as depicted in Figure 3. The framework consists of several key steps, which are summarized as follows:

PB Data Processing: The PB data from the samples are propagated and aggregated using a graph neural network. This network captures the relationships and dependencies between features, enabling the extraction of essential information. Subsequently, a graph pooling method is applied to select crucial features and characterize the information, resulting in the generation of the corresponding vector, denoted as $z_{a}$.

CT Data Processing: The CT data from the samples are processed using a 3D convolutional neural network. This network effectively extracts features from the images, generating the corresponding vector, denoted as $z_{v}$.

Multi-modal Fusion: To combine the vectors obtained from different modalities, we utilize the Low-rank Multi-modal Fusion method. This fusion technique integrates the information from the PB and CT data while reducing noise and redundancy.

Information Interaction and Adaptation: To capture the interaction between different modalities and facilitate information adaptation, we employ the Cross-modal Transformer. Specifically, we use the Cross-modal Transformer to characterize each modality separately. This results in the characterization of $z_{a}$ and $z_{v}$ using Cross-modal Transformer, respectively, leading to the generation of corresponding feature vectors. These feature vectors are then fed into the Transformer module with Self-Attention, allowing for the extraction of final feature vectors.

Final Integration: The final feature vectors obtained from the previous step are stitched together, representing the comprehensive fusion of information from different modalities. This integrated representation encompasses the combined knowledge from both the PB and CT data.

Importantly, our proposed method can be easily extended to accommodate additional modal data. Furthermore, during the pooling process, efficient indicators can be performed to identify the most informative features that characterize the data.

### 3.2. GNN for Processing Physiological and Biochemical Data

In this subsection, we present a method for processing physiological and biochemical data using graph neural networks, which can also be applied to various histological data. Graph neural networks are chosen for their powerful capabilities in information representation and capturing relationships. The first step is to construct the graph structure that represents the relational data. In the experiments conducted in this paper, the physiological and biochemical data are processed by calculating the Pearson Correlation Coefficient. This coefficient is used to establish the correspondence between samples, creating the foundation for constructing the graph structure. Specifically, the correlation coefficients provide valuable information about the relationships and dependencies between different physiological and biochemical measurements.

If the method is applied to process other types of data, such as genomics or proteomics data, characteristic relational networks from resources such as GeneMANIA [21] and String [22] can be utilized to construct the graph structure. These resources provide valuable insights into the functional associations and interactions between genes or proteins, enabling the construction of an informative graph structure that captures the underlying relationships in the data.

In our approach, we represent the features in the original data as nodes in a graph. The initial vectors of these nodes are derived from the corresponding sample expression values. Specifically, we define a set of node sets $V=v_{1},\;v_{2},\;\ldots ,\;v_{N}$ and a set of edge connections $E=e_{1},\;e_{2},\;\ldots ,\;e_{M}$, where nodes represent the features and edges represent the relationships between these features. To establish edges, we consider all similarities with absolute correlation values greater than 0.3.

For each node $v_{i}$, its initial vector is denoted as $h_{v_{i}}^{0}=X_{i1},\;X_{i2},\;X_{i3},\;\ldots ,\;X_{iN}$, where $X_{ij}$ represents the expression value of the *i*-th feature in the original data for the *j*-th sample.

To update the hidden state vector of a node, we employ an aggregation function, as shown in Equation (3). This function allows us to incorporate information from the first-order neighborhood, denoted as *N*, of any given node. By aggregating the information from neighboring nodes, the hidden state vector of each node is updated to capture relevant relationships and dependencies within the graph structure. This process enables the graph neural network to update and refine the representations of the features based on their relationships with other features. The aggregation function plays a crucial role in capturing the information flow and relationships within the graph, facilitating effective information representation and learning.

(3) $$h_{N\left(v_{i}\right)}^{K}\leftarrow \;\mathrm{AGGREGATE}{\;}_{K}\left(\left\{h_{N\left(v_{i}\right)}^{K-1},\forall v_{i}\in N\left(v_{i}\right)\right\}\right)$$

where AGGREGATE ${}_{K}\left(*\right)$ denotes the *K* layer’s aggregation function, we use average aggregation. Subsequently, the node vectors are stitched together, and the hidden state vectors are updated by normalizing the vectors using Equations (4) and (5).

(4) $$h_{v_{i}}^{k}\leftarrow \sigma \left(W^{k}\cdot \mathrm{COUNCAT}\left(h_{v_{i}}^{k-1},h_{N\left(v_{i}\right)}^{k}\right)\right)$$

(5) $$h_{v_{i}}^{k}\leftarrow h_{v_{i}}^{k}/{∥h_{v_{i}}^{k}∥}_{2},\;v_{i}\in v$$

where $W^{k}$ denotes the parameter matrix of the *K*-th layer, indicating the splicing operation. To perform feature selection and identify the most informative features, as well as to obtain an effective information representation, we employ a TopK-based graph pooling method. This pooling method allows us to select the most relevant nodes and discard redundant features. A detailed description of this method is provided later in this chapter. After obtaining the pooled set of nodes, we utilize a readout layer to aggregate global information from the graph. This aggregation involves stitching together the average pooled information and the maximum pooled information at different scales. These aggregated features are then summed up to form the final global information representation.

The process of aggregating global information can be described using Equations (6) and (7). These equations outline the specific calculations involved in combining the average pooled and maximum pooled information to obtain the final global representation.

(6) $$S^{\left(l\right)}=\frac{1}{N}\sum_{i=0}^{N}x_{i}∥\max_{i}$$

(7) $$s=\sum_{l=1}^{L}s^{\left(l\right)}$$

By applying this readout layer, we effectively capture and summarize the global information within the graph structure. This information serves as a comprehensive representation of the underlying relationships and dependencies among the features, providing valuable insights for subsequent analysis and classification tasks.

In the experiments presented in this study, the GNN model comprises four convolutional layers for node information propagation and aggregation. Each of these layers is followed by a ReLU activation function and is regularized using Dropout. This is succeeded by a TopKpooling layer responsible for node pooling and outputting node scores. The pooled nodes are processed by a linear layer to extract features, resulting in a 64-dimensional vector that serves as the extracted feature, acting as the input for multi-modal information. Moreover, during the GNN training, a linear layer is positioned at the end, performing classification tasks. To elaborate, the GNN encompasses two linear layers: the initial layer extracts a 64-dimensional feature vector, while the concluding layer caters to the classification task. The network is designed to minimize cross-entropy loss, optimized using Adam, and the network parameters are initialized via the Xavier method.

### 3.3. 3DCNN for Processing Image Data

Two-dimensional convolutional neural networks, such as ResNet, have shown remarkable progress in various image-related tasks, especially in image classification. ResNet incorporates shortcut connections that enable signals to bypass individual layers and proceed to the subsequent layers in the sequence. This design facilitates the training of deep networks by allowing the gradient flow to propagate from later layers to earlier layers, mitigating the vanishing gradient problem [23].

Considering the 3D nature of the patient’s CT image data, our proposed method utilizes a ResNet-based 3D convolutional network (3DCNN) to capture the inter-layer information present in the CT images. This choice aims to enhance feature extraction by effectively modeling the volumetric features and dependencies within the CT images, leading to more accurate and comprehensive representations [24].

In our approach, we employ ResNet50 3D as the processing network for the image data, retaining the main structure of the network without modifications. However, to ensure the consistency of the output dimensions with other modalities, we add a fully connected layer at the end of the network to control the output feature dimension, aligning it with the dimensions of other modalities. To optimize the network parameters effectively, we first pre-train the network using all the available image data. Since the number of CT image samples is typically larger than the physiological and biochemical data samples, this pre-training step ensures the network captures relevant features from the image modality. Subsequently, during the multi-modal fusion process, we update the network parameters to fine-tune the model.

During training, the network is fed with input images of size 30 × 224 × 224, and the batch size is set to 10. The Adam optimizer is utilized, with a learning rate of $2\times 10^{-5}$, betas = (0.9, 0.999), eps = $1\times 10^{-8}$, and weight_decay = 0.001. The learning rate is dynamically adjusted during model training, reducing it by a factor of 0.1 every ten epochs. The cross-entropy loss function is employed to train the model. By following this training strategy and utilizing the ResNet-based 3D convolutional network, we ensure effective processing of the CT image data, allowing the network to learn discriminative features and improve the overall performance of our proposed method.

### 3.4. Multi-modal Fusion Method

In the proposed method, we perform fusion of the obtained multi-modal data vectors using the low-rank matrix factorization method. This method employs a low-rank decomposition factor for tensor fusion, which helps address the high-dimensional problem that arises when directly fusing tensors. Specifically, we assume that the weight vector to be learned is represented as *W*, and we consider *W* as a set of M-rank vectors, denoted as ${\bar{W}}_{k}\in R^{d_{1}\times \ldots \times d_{M}}$, where *k* ranges from 1 to $d_{h}$. Each ${\bar{W}}_{k}$ can be obtained through the decomposition described in Equation (8).

(8) $${\bar{W}}_{k}=\sum_{i=1}^{R}\overset{M}{\underset{m=1}{⨂}}w_{m,k}^{\left(i\right)},\;w_{m,k}^{\left(i\right)}\in R_{m}^{d}$$

The rank of the tensor, denoted as R, represents the minimum value that ensures a valid decomposition. The vectors $w_{m,k}^{\left(i\right)}\left(m=1\ldots M,\;i=1\ldots R\right)$ in the set serve as the decomposition factors for reconstructing the low-rank $\bar{W}k$ from the original vector *R*. These vectors can be combined to form M mode-specific low-rank factors. Specifically, $wm^{\left(i\right)}=\left[w{m,1}^{\left(i\right)},\;w{m,2}^{\left(i\right)},\;\ldots ,\;w_{m,d_{h}}^{\left(i\right)}\right]$ represents the low-rank factor corresponding to mode-m, and ${\left\{w_{m}^{\left(i\right)}\right\}}_{i=1}^{r}$ represents the collection of such low-rank factors. By utilizing Equation (9), we can obtain the low-rank weight tensor.

(9) $$W=\sum_{i=1}^{r}\overset{M}{\underset{m=1}{⨂}}w_{m}^{\left(i\right)}$$

According to the above definition, assuming that the *M* modal vectors we want to fuse are $Z={⨂}_{m=1}^{M}z_{m}$, the fused vector *h* is calculated as shown in Equation (10).

(10) $$\begin{array}{cc} h & =\left(\sum_{i=1}^{r}\overset{M}{\underset{m=1}{⨂}}w_{m}^{\left(i\right)}\right)\cdot Z=\sum_{i=1}^{r}\left(\overset{M}{\underset{m=1}{⨂}}w_{m}^{\left(i\right)}\cdot Z\right)\\ \; & =\sum_{i=1}^{r}\left(\overset{M}{\underset{m=1}{⨂}}w_{m}^{\left(i\right)}\cdot \overset{M}{\underset{m=1}{⨂}}z_{m}\right)\\ \; & =\overset{M}{\underset{m=1}{⋀}}\left[\sum_{i=1}^{r}w_{m}^{\left(i\right)}\cdot z_{m}\right] \end{array}$$

where $\overset{M}{\underset{m=1}{⋀}}$ is denoted as the product of the elements of a series of vectors. In this paper, we fuse two modal vectors, which are assumed to be denoted as $Z=z_{a},z_{v}$, respectively, and their fusion vector *h* is represented as shown in Equation (11).

(11) $$\begin{array}{cc} h & =\left(\sum_{i=1}^{r}w_{a}^{\left(i\right)}⊗w_{v}^{\left(i\right)}\right)\cdot Z\\ \; & =\left(\sum_{i=1}^{r}w_{a}^{\left(i\right)}\cdot z_{a}\right)\circ \left(\sum_{i=1}^{r}w_{v}^{\left(i\right)}\cdot z_{v}\right) \end{array}$$

After obtaining the fusion vector *h*, we employ a combination of Transformer modules with Cross-modal Attention and Transformer modules with Self-Attention to further fuse the vectors. After obtaining the fusion vector, we employ a combination of Transformer and Cross-modal Transformer to further enhance the fusion of vectors. Specifically, the Transformer is underpinned by the ‘Self-Attention Mechanism’ to discern dependencies within a sequence. With this mechanism in place, the Transformer can process an entire sequence in parallel, bolstering efficiency. It adeptly captures various dependencies within sequences, proving especially efficacious for fusing information from diverse modalities. The attention mechanism integral to the Transformer is articulated as presented in Equation (12).

(12) $$\mathrm{Attention}\left(Q,K,V\right)=\mathrm{softmax}\left(\frac{QK^{T}}{\sqrt{d_{k}}}\right)V$$

In this context, Q, K, and *V* symbolize the query, key, and value, respectively, while $d_{k}$ denotes the dimensionality of the key. The Cross-modal Transformer, a nuanced variant of the Transformer, is tailored explicitly for processing information emanating from disparate modalities, such as text and images. This archetype proves invaluable for amalgamating data streams like gene expression profiles and medical imaging data. Leveraging its specialized attention mechanism, the Cross-modal Transformer can intuit and incorporate semantic connections spanning different modalities, facilitating the processing of multi-modal data within a unified paradigm. To synthesize multi-modal data, the Cross-modal Transformer utilizes an attention formula delineated in Equation (13).

(13) $$Cross-Attention\left(Q_{m},K_{n},V_{n}\right)=\mathrm{softmax}\left(\frac{Q_{m}K_{n}^{T}}{\sqrt{d_{k}}}\right)V_{n}$$

Herein, $Q_{m}$ is derived from modality *m*, whereas $K_{n}$ and $V_{n}$ are sourced from modality *n*. This architecture endows the model with the capacity to extract vectors from modality *n* that resonate with modality *m*. Detailed explanations of these two modules can be found in the literature [17,25], and we have integrated these two methods for multi-modal data fusion. To illustrate, let us consider two modal data, namely $z_{a}$ and $z_{v}$. First, the vectors from $z_{a}$ and $z_{v}$ are passed through the LMF layer to obtain the fusion vector *h*. Then, each modality’s vector and the fusion vector are fed into the corresponding Cross-modal Transformer module to obtain the cross-modal vector representation. Finally, these vectors pass through one layer of the Transformer and are concatenated to produce the final vectors. The classification task is performed using a Multilayer Perceptron (MLP). The proposed method is designed as an end-to-end framework, where all networks are jointly trained, the pre-trained network parameters are updated, and cross-entropy loss is used as the loss function in the experiments.

### 3.5. GNN Identifies PB Indicators

In order to perform feature selection on the graph and identify the essential PB indicators, we utilize a TopK-based graph pooling method. This method allows us to downsample the nodes of the entire graph to a reduced set of kN nodes, where *k* is a superparameter representing the pooling rate, with k∈(0,1). The downsampling process is based on the learned node importance values, denoted as *z*, which are calculated using Equation (14).

(14) $$X^{\prime }=X_{i,:}$$

(15) i=top−rank(z,kN)

where $X_{i,:}$ denotes the slice-by-row operation of the original feature matrix according to the value of vector *i*. In Equation (15), the importance *z* of a node is calculated as shown in Equation (16).

(16) $$z=\frac{Xp}{∥p∥}$$

In this method, the importance ranking of nodes is determined based on the size of the projection, which is obtained by projecting the node’s feature vector onto the global basis vector *p*. The projection size serves as a gradient threshold, with nodes having smaller projections indicating minor gradient information. The L2 parametrization, denoted as ||∗||, is used to calculate the projection size.

## 4. Experimental Results

In this section, we conducted experimental validation of the proposed method on the COPD multi-modal dataset. First, we analyzed the performance of using single-modal and multi-modal data to demonstrate the necessity of multi-modal research. Subsequently, we conducted ablation and comparison experiments to illustrate the necessity of each module in our proposed approach. Finally, we performed statistical tests on the selected features in the physiological and biochemical data to demonstrate their effectiveness.

### 4.1. Performance of the Proposed Method on Multi-modal Data

In this subsection, we evaluate the performance of the proposed method on both single-modal and multi-modal data. We use six metrics, namely Accuracy (Acc), Precision (Pre), Sensitivity (Sen), Specificity (Spe), F1-Score (F1), and AUC, to assess the model’s performance.

For the single-modal experiments, we utilize the PB data for feature pooling and characterization. The classification task is performed using our proposed GNN model, and the loss function used is cross-entropy. In addition, we apply the CT data to the ResNet50 3D network, and the obtained feature vectors are classified using MLP. Again, cross-entropy loss is employed as the loss function.

**Figure 4:**
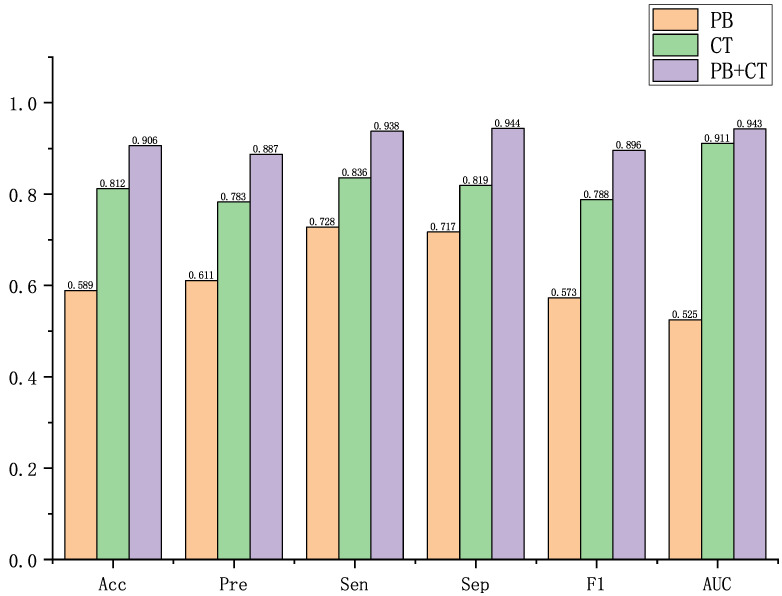
Performance of the proposed method with single and multi-modal data, where PB denotes Physiology and Biochemistry data and CT denotes Computed Tomography data.

In the multi-modal fusion experiments, we perform end-to-end training using our proposed method. The classification task is conducted using the same MLP, and the loss function remains cross-entropy. The detailed results of these experiments can be found in Figure 4.

From the results presented in Figure 4, it can be observed that the introduction of multi-modal data can improve performance across various evaluation metrics compared to using single-modal data as a baseline. Specifically, the fusion model achieved improvements of 52.82%, 45.17%, 28.85%, 31.66%, 56.37%, and 79.62% over the Physiology and Biochemistry data alone in terms of Accuracy, Precision, Sensitivity, Specificity, F1-Score and AUC, respectively. In comparison to the Computed Tomography (CT) data alone, the fusion model exhibited enhancements of 11.58%, 13.28%, 12.20%, 15.26%, 13.71%, and 3.51% in the same evaluation metrics. These results highlight the importance and necessity of leveraging multi-modal data for disease classification, as well as the effectiveness of the multi-modal fusion method proposed in our approach.

### 4.2. Comparison of Different Expression Data Processing Methods

To assess the effectiveness of the expressive data processing methods utilized in our proposed approach, we conducted a comparison with several feature selection methods, including L1 regularization (Lasso) [26], L2 regularization (Ridge) [27], correlation coefficient (Corr) [28], decision tree (DT) [29], and random forest (RF) [30]. The comparison was performed on both unimodal and multi-modal data. For the unimodal data, different feature selection methods were used for comparison, and classification was subsequently conducted using MLP. However, for our proposed GNN method, given its inherent capability to support both feature selection and classification, there was no need to employ MLP for the final classification task. Detailed results for this section are presented in Table 2, where FS denotes feature selection methods and CLF indicates the classification model. For multi-modal data, the comparative methods were incorporated into our proposed framework for processing PB data. That is, we employed the methods from Table 3 for feature selection of PB data and utilized MLP for feature extraction. However, as the proposed GNN method inherently supports feature extraction, the MLP was rendered unnecessary. In Table 3, FS stands for feature selection methods, and CLF represents the classifier employed for feature extraction.

For the unimodal data, each method was employed as a feature selection technique, and the subsequent classification was conducted using a MLP model. All methods were tuned to select the same number of features. Regarding multi-modal fusion, we employed these methods as data processing techniques for PB data, and the feature vectors were then characterized using MLP. The results of the experiments on the single modality are presented in Table 2, while the results for multi-modal data can be found in Table 3.

From the results presented in Table 2, it is evident that the expressive data processing methods employed in our approach outperformed the compared methods across various metrics. The GNN method demonstrated superior performance compared to the other methods in terms of metrics other than classification accuracy. While the RF method achieved higher accuracy in classification, the GNN method consistently outperformed it in other evaluation metrics. Moreover, we analyzed the performance of the GNN model on the validation set. The corresponding metrics across these evaluation parameters were 0.586, 0.610, 0.717, 0.721, 0.583, and 0.584. When juxtaposed against the results in Table 2, it is evident that the GNN model is not prone to overfitting. Moving on to Table 3, we observe that although the RF method achieved higher classification accuracy on individual modalities, the GNN method outperformed it in the overall fusion model. This can be attributed to the end-to-end training and the ability to control the pooling process offered by the GNN method, which is challenging to achieve with traditional feature selection methods.

### 4.3. Comparison of Different CT Data Processing Methods

In order to assess the effectiveness of the CT data processing methods employed in our proposed approach, we conducted a comparison with several image processing methods, namely the Low Attenuation Area (LAA) method [31], the Local Binary Patterns (LBP) method [32], the VGG16 method [33], and the DCT-MIL method [34]. These methods were evaluated in both single-modal and multi-modal fusion models for image classification performance. We replaced the image processing methods with the respective image feature extraction networks and standardized the output vector dimension using a fully connected layer. The results of the experiments conducted on single modality are presented in Table 4, while the results on multi-modal data are shown in Table 5.

The results in Table 4 demonstrate that the ResNet 50 3D used in our proposed method outperforms most of the compared methods in terms of image classification performance. This is attributed to its superior capability of capturing interlayer information in the images. Although VGG16 achieves better performance in terms of Sensitivity, ResNet outperforms it in the other evaluation metrics. Turning to Table 5, ResNet maintains its lead among the compared methods when serving as an image feature extractor in the multi-modal fusion framework. Additionally, it is worth noting that the texture feature extraction-based methods do not perform as well as the deep network-based methods, suggesting the advantage of deep networks in capturing complex image representations.

### 4.4. Comparison of Different Multi-Modal Fusion Methods

Table 6 displays the comparison results of different multi-modal fusion methods, including vector concatenation, max-pooling, mean-pooling, TFN, LMF, Transformer, and the proposed LMF+Transformer method.

Comparing the LMF and Transformer methods, both approaches outperform the simple fusion methods. LMF performs better than vector concatenation, max-pooling, and mean-pooling, highlighting the significance of low-rank matrix factorization in feature fusion. Transformer also shows competitive performance, emphasizing the effectiveness of self-attention mechanisms for capturing interactions between different modalities.

Among all the methods, the proposed LMF + Transformer method demonstrates the best overall performance, achieving the highest accuracy, Sensitivity, Specificity, F1-Score, and AUC. This indicates that combining low-rank matrix factorization with Transformer-based fusion leads to superior multi-modal data integration and classification performance.

The results provide strong evidence for the superiority of the proposed multi-modal fusion method and highlight the limitations of simple fusion methods. The LMF + Transformer method shows promising potential for enhancing disease classification and feature selection tasks.

### 4.5. Analysis of Indicators Selected by the Proposed Method

In this subsection, we analyze the indicators selected by the proposed method. Among all the physiological and biochemical indicators, we identified the four most important indicators using the proposed method. The selection process involved initial pooling for filtering and subsequent sequential removal of each indicator to observe the corresponding decrease in model performance. We considered indicators that exhibited a larger decrease in model performance as more important.

**Figure 5:**
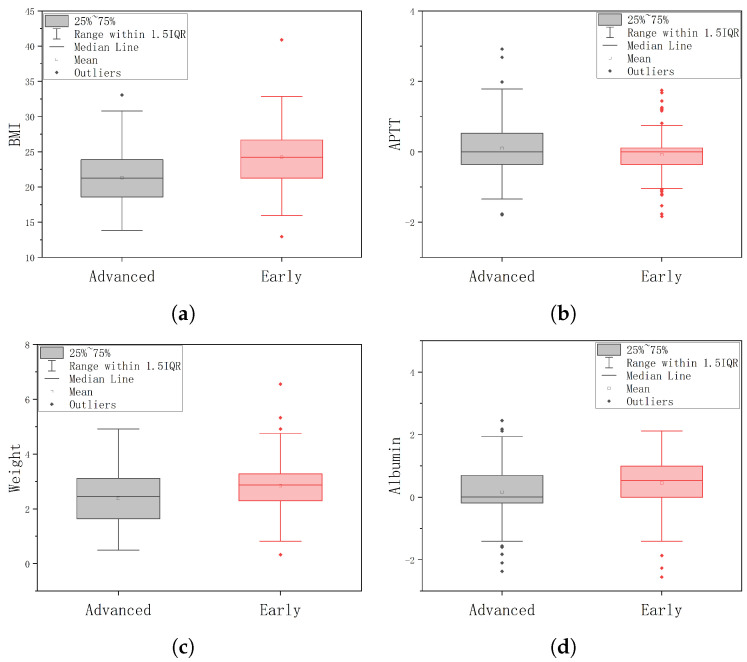
The expression distribution of the four most important indicators selected by the proposed method in the Early and Advanced groups, (**a**) represents the BMI indicator, (**b**) represents the APTT indicator, (**c**) represents the Weight indicator, and (**d**) represents the Albumin indicator.

The four selected indicators are BMI, Upon activation of partial thromboplastin (APTT), Weight, and Albumin. These indicators were chosen based on their significance in differentiating between the Early and Advanced groups. We normalized the indicators using the z-score and displayed their expression distributions in the two groups, as shown in Figure 5. The distribution of these indicators exhibits significant differences between the Early and Advanced groups, suggesting their potential as valuable indicators for disease classification.

We also conducted statistical analyses to assess the significance of these characteristics between the Early and Advanced groups. Independent samples *t*-tests were performed to compare the mean values of each indicator in the two groups, as shown in Table 7.

In the BMI group, the mean value of the Early group was significantly higher than that of the Advanced group, with a statistically significant difference of 0.721 (0.504–0.937) between the two groups (t = 6.550, *p* < 0.001). In the APTT group, the mean value of the Early group was significantly lower than that of the Advanced group, with a difference of −0.251 (−0.481–0.021) between the two groups, and the difference was statistically significant (t = −2.150, *p* = 0.032). In the Weight group, the mean value of the Early group was significantly higher than that of the Advanced group, with a difference of 0.446 (0.22–0.672) between the two groups, and the difference was statistically significant (t = 3.886, *p* < 0.001). In the Albumin group, the mean value of the Early group was significantly higher than that of the Advanced group, with a difference of 0.16 (0.049–0.272) between the two groups, and the difference was statistically significant (t = 2.836, *p* < 0.005).

These statistical analyses further support the significant differences observed in the distribution of these indicators between the Early and Advanced groups.

## 5. Conclusions

In this paper, a novel multi-modal fusion method is proposed for processing biomedical data. The method utilizes graph neural networks and 3D convolutional neural networks to process PB data and CT data, respectively, extracting relevant features from each modality. The fusion process incorporates Low-rank Multi-modal Fusion and Multi-modal Transformer-based fusion, enabling automatic learning of inter-modal relationships.

The effectiveness and superiority of the proposed method are demonstrated through experiments on lung CT imaging data and physiological and biochemical data from patients with Chronic Obstructive Pulmonary Disease. The method outperforms various alternative approaches, showcasing its capability in handling multi-modal biomedical data.

Furthermore, the proposed method enables the selection of validated indicators and offers flexibility for extension to accommodate data from additional modalities. Overall, the method presents a promising solution for multi-modal data analysis and fusion in the field of biomedical research.

## Figures and Tables

**Table 1:** Biochemical indicators.

Inspection Items	Indicators	Abbreviation	Unit
Routine blood test	White blood cell	WBC	$10^{9}$/L
	Neutrophil percentage	NEUT	%
	Hemoglobin	HGB	%
	Thrombocytopenia	PCT	%
	Platelet count	PLT	$10^{9}$/L
Liver function	Albumin	ALB	g/L
	Aspartate aminotransferase	AST	U/L
	Alanine aminotransferase	ALT	U/L
	Total bilirubin	TBIL	umol/L
	Creatinine	CREA	umol/L
	Creatine kinase	CK	U/L
	Creatine kinase MB isoenzyme	CKMB	U/L
	Lactate dehydrogenase	LDH	U/L
CRP	C-reactive protein	CRP	mg/L
Coagulation five	Prothrombin time	PT	seconds
	Upon activation of partial thromboplastin	APTT	seconds
	Dimers	DD_D	ug/L

**Table 2:** The proposed PB data processing method compares the results with other methods on single modal data. Bolded indicates optimal results and underlined indicates suboptimal results.

FS Method	CLF	Acc	Pre	Sep	Sep	F1	AUC
Lasso [26]	MLP	0.523	0.594	0.711	0.699	0.504	0.512
Ridge [27]	MLP	0.554	0.603	0.717	0.711	0.523	0.518
Corr [28]	MLP	0.576	0.605	0.721	0.713	0.554	0.531
DT [29]	MLP	0.568	0.598	0.715	0.713	0.548	0.552
RF [30]	MLP	**0.591**	0.608	0.726	0.707	0.566	0.553
GNU(Ours)	GNN	0.589	**0.611**	**0.728**	**0.717**	**0.573**	**0.567**

**Table 3:** The results of the proposed PB data processing methods in multi-modal fusion are compared with other methods with which we replaced the expression data feature extraction process in the fusion framework, respectively. Bolded indicates optimal results, and underlined indicates suboptimal results.

FS Method	CLF	Acc	Pre	Sep	Sep	F1	AUC
Lasso [26]	MLP	0.866	0.845	0.895	0.877	0.867	0.838
Ridge [27]	MLP	0.879	0.843	0.912	0.898	0.866	0.856
Corr [28]	MLP	0.868	0.856	0.915	0.901	0.871	0.870
DT [29]	MLP	0.862	0.853	0.921	0.922	0.869	0.866
RF [30]	MLP	0.889	0.860	0.924	0.931	0.877	0.914
GNN(Ours)	GNN	**0.906**	**0.887**	**0.938**	**0.944**	**0.896**	**0.943**

**Table 4:** The proposed CT data processing method compares the results with other methods on single modal data. Bolded indicates optimal results and underlined indicates suboptimal results.

Method	Acc	Pre	Sep	Sep	F1	AUC
LAA [31]	0.778	0.765	0.768	0.742	0.773	0.704
LBP [32]	0.683	0.734	0.762	0.734	0.680	0.699
VGG16 [33]	0.798	0.756	**0.838**	0.809	0.781	0.821
DCT-MIL [34]	0.621	0.608	0.612	0.617	0.611	0.593
ResNet 50 3D (Ours)	**0.812**	**0.783**	0.836	**0.812**	**0.788**	**0.822**

**Table 5:** The results of the proposed CT data processing methods in multi-modal fusion are compared with other methods with which we replaced the image data feature extraction process in the fusion framework, respectively. Bolded indicates optimal results, and underlined indicates suboptimal results.

Method	Acc	Pre	Sep	Sep	F1	AUC
LAA [31]	0.821	0.813	0.866	0.856	0.822	0.811
LBP [32]	0.808	0.782	0.853	0.862	0.813	0.798
VGG16 [33]	0.900	0.865	0.921	0.911	0.890	0.922
DCT-MIL [34]	0.788	0.738	0.809	0.709	0.794	0.697
ResNet 50 3D (Ours)	**0.906**	**0.887**	**0.938**	**0.944**	**0.896**	**0.943**

**Table 6:** Comparison results of multi-modal data fusion method of the proposed method with other multi-modal data fusion methods. Bolded indicates optimal results, and underlined indicates suboptimal results.

Method	Acc	Pre	Sep	Sep	F1	AUC
Concatenation	0.877	0.850	0.919	0.921	0.877	0.868
Max-pool	0.871	0.847	0.914	0.900	0.866	0.857
Mean-pool	0.870	0.849	0.919	0.917	0.868	0.859
TFN	0.879	0.871	0.920	0.922	0.874	0.892
LMF	0.881	0.872	0.921	0.913	0.878	0.917
TF	0.892	0.879	**0.941**	0.927	0.884	0.933
LMF + TF (Ours)	**0.906**	**0.887**	0.938	**0.944**	**0.896**	**0.943**

**Table 7:** Statistical differences in physiological and biochemical features in the Early and Advanced groups.

Features	Difference	t-Value	*p*-Value
BMI	0.721	6.55	<0.001
APTT	−0.251	−2.15	=0.032
Weight	0.446	3.886	<0.001
Albumin	0.16	2.836	<0.005

## Data Availability

The data that support the findings of this study are available from the corresponding author upon reasonable request.

## References

[1] Singh A., Shannon C.P., Gautier B., Rohart F., Vacher M., Tebbutt S.J., Lê Cao K.A. (2019). DIABLO: An integrative approach for identifying key molecular drivers from multi-omics assays. Bioinformatics.

[2] McCabe S.D., Lin D.Y., Love M.I. (2020). Consistency and overfitting of multi-omics methods on experimental data. Brief. Bioinform..

[3] Peng J., Zhu X., Wang Y., An L., Shen D. (2019). Structured sparsity regularized multiple kernel learning for Alzheimer’s disease diagnosis. Pattern Recognit..

[4] Park M.-K., Lim J.-M., Jeong J., Jang Y., Lee J.-W., Lee J.-C., Kim H., Koh E., Hwang S.-J., Kim H.-G. (2022). Deep-Learning Algorithm and Concomitant Biomarker Identification for NSCLC Prediction Using Multi-Omics Data Integration. Biomolecules.

[5] Chen R.J., Lu M.Y., Wang J., Williamson D.F.K., Rodig S.J., Lindeman N.I., Mahmood F. (2020). Pathomic fusion: An integrated framework for fusing histopathology and genomic features for cancer diagnosis and prognosis. IEEE Trans. Med. Imaging.

[6] Günther O.P., Chen V., Freue G.C., Balshaw R.F., Tebbutt S.J., Hollander Z., Takhar M., McMaster W.R., McManus B.M., Keown P.A. (2012). A computational pipeline for the development of multi-marker bio-signature panels and ensemble classifiers. BMC Bioinform..

[7] Sun Y., Goodison S., Li J., Liu L., Farmerie W. (2007). Improved breast cancer prognosis through the combination of clinical and genetic markers. Bioinformatics.

[8] Wang B., Mezlini A.M., Demir F., Fiume M., Tu Z., Brudno M., Haibe-Kains B., Goldenberg A. (2014). Similarity network fusion for aggregating data types on a genomic scale. Nat. Methods.

[9] Van De Wiel M.A., Lien T.G., Verlaat W., van Wieringen W.N., Wilting S.M. (2016). Better prediction by use of co-data: Adaptive group-regularized ridge regression. Stat. Med..

[10] Ning Z., Pan W., Chen Y., Xiao Q., Zhang X., Luo J., Wang J., Zhang Y. (2020). Integrative analysis of cross-modal features for the prognosis prediction of clear cell renal cell carcinoma. Bioinformatics.

[11] Jl A., Iyc B., Chj C. (2020). An efficient multivariate feature ranking method for gene selection in high-dimensional microarray data. Expert Syst. Appl..

[12] Saranya G., Pravin A. (2022). A novel feature selection approach with integrated feature sensitivity and feature correlation for improved prediction of heart disease. J. Ambient. Intell. Humaniz. Comput..

[13] Huang Z., Zhan X., Xiang S., Johnson T.S., Helm B., Yu C.Y., Zhang J., Salama P., Rizkalla M., Han Z. (2019). SALMON: Survival analysis learning with multi-omics neural networks on breast cancer. Front. Genet..

[14] Wang T., Shao W., Huang Z., Tang H., Zhang J., Ding Z., Huang K. (2021). MOGONET integrates multi-omics data using graph convolutional networks allowing patient classification and biomarker identification. Nat. Commun..

[15] Deligani R.J., Borgheai S.B., McLinden J., Shahriari Y. (2021). Multi-modal fusion of EEG-fNIRS: A mutual information-based hybrid classification framework. Biomed. Opt. Express.

[16] Sahay S., Okur E., Kumar S.H., Nachman L. (2020). Low rank fusion based transformers for multi-modal sequences. arXiv.

[17] Tsai Y.H.H., Bai S., Liang P.P., Kolter J.Z., Morency L.P., Salakhutdinov R. (2019). Multi-modal transformer for unaligned multi-modal language sequences. Proceedings of the 57th Annual Meeting of the Association for Computational Linguistics.

[18] Xie W., Li W., Zhang S., Wang L., Yang J., Zhao D. (2022). A novel biomarker selection method combining graph neural network and gene relationships applied to microarray data. BMC Bioinform..

[19] Yu K., Xie W., Wang L., Zhang S., Li W. (2021). Determination of biomarkers from microarray data using graph neural network and spectral clustering. Sci. Rep..

[20] Hofmanninger J., Prayer F., Pan J., Röhrich S., Prosch H., Langs G. (2020). Automatic lung segmentation in routine imaging is primarily a data diversity problem, not a methodology problem. Eur. Radiol. Exp..

[21] Warde-Farley D., Donaldson S.L., Comes O., Zuberi K., Badrawi R., Chao P., Franz M., Grouios C., Kazi F., Lopes C.T. (2010). The GeneMANIA prediction server: Biological network integration for gene prioritization and predicting gene function. Nucleic Acids Res..

[22] Damian S., Andrea F., Stefan W., Kristoffer F., Davide H., Jaime H.C., Milan S., Alexander R., Alberto S., Tsafou K.P. (2015). STRING v10: Protein–protein interaction networks, integrated over the tree of life. Nucleic Acids Res..

[23] He K., Zhang X., Ren S., Sun J. Deep Residual Learning for Image Recognition. Proceedings of the 2016 IEEE Conference on Computer Vision and Pattern Recognition (CVPR).

[24] Hara K., Kataoka H., Satoh Y. Can spatiotemporal 3d cnns retrace the history of 2d cnns and imagenet?. Proceedings of the IEEE Conference on Computer Vision and Pattern Recognition.

[25] Vaswani A., Shazeer N., Parmar N., Uszkoreit J., Jones L., Gomez A.N., Kaiser Ł., Polosukhin I. (2017). Attention is all you need. Adv. Neural Inf. Process. Syst..

[26] Muthukrishnan R., Rohini R. (2016). LASSO: A feature selection technique in predictive modeling for machine learning. Proceedings of the 2016 IEEE International Conference on Advances in Computer Applications (ICACA).

[27] Xu W., Liu X., Leng F., Li W. (2020). Blood-based multi-tissue gene expression inference with Bayesian ridge regression. Bioinformatics.

[28] Li Y., Dai Z., Cao D., Luo F., Chen Y., Yuan Z. (2020). Chi-MIC-share: A new feature selection algorithm for quantitative structure–activity relationship models. RSC Adv..

[29] Zhou H., Zhang J., Zhou Y., Guo X., Ma Y. (2021). A feature selection algorithm of decision tree based on feature weight. Expert Syst. Appl..

[30] Zhou Q., Zhou H., Li T. (2016). Cost-sensitive feature selection using random forest: Selecting low-cost subsets of informative features. Knowl.-Based Syst..

[31] Sorensen L., Shaker S.B., de Bruijne M. (2010). Quantitative Analysis of Pulmonary Emphysema Using Local Binary Patterns. IEEE Trans. Med. Imaging.

[32] Kaplan K., Kaya Y., Kuncan M., Ertunç H.M. (2020). Brain tumor classification using modified local binary patterns (LBP) feature extraction methods. Med. Hypotheses.

[33] Simonyan K., Zisserman A. (2014). Very deep convolutional networks for large-scale image recognition. arXiv.

[34] Xu C., Qi S., Feng J., Xia S., Kang Y., Yao Y., Qian W. (2020). DCT-MIL: Deep CNN transferred multiple instance learning for COPD identification using CT images. Phys. Med. Biol..

